# Biofilm Formation on Invasive Medical Devices in Adult Intensive Care Units: A Scoping Review of Biomedical and Clinical Evidence

**DOI:** 10.3390/jcm15197388

**Published:** 2026-09-23

**Authors:** Valeria Lizbeth Apolo-Aguilar, Lennys Beatriz Berutti-Suarez, José Luis Muñoz Carrillo

**Affiliations:** 1 Dirección de Posgrado, Universidad Politécnica Estatal del Carchi, Tulcán 040101, Ecuador; valeria.apolo@upec.edu.ec; 2 Laboratorio de Inmunología, Centro Universitario de los Lagos, Universidad de Guadalajara, Lagos de Moreno 47460, Mexico; 3 Centro de Investigación e Innovación Glogal University (CIIGU), Aguascalientes 20116, Mexico

**Keywords:** biofilms, intensive care units, catheters, ventilator-associated pneumonia, drug resistance, microbial, critical care, infection control, device-associated infections, healthcare-associated infections, scoping review

## Abstract

**Background/Objectives:** Biofilm formation on invasive medical devices represents a major challenge in intensive care units (ICUs), contributing to device-associated infections, antimicrobial resistance, and adverse clinical outcomes. Despite its clinical relevance, the extent and characteristics of the available evidence on biofilm formation in invasive medical devices used in adult ICUs remain heterogeneous and incompletely mapped. The objective was to map and characterize the available evidence on biofilm formation in invasive medical devices used in adult ICUs. **Methods**: A scoping review was conducted following PRISMA-ScR guidelines. The review was structured using the Population–Concept–Context (PCC) framework. A comprehensive search was performed in PubMed, Scopus, Web of Science, Embase, and ScienceDirect. Primary studies evaluating biofilm formation on invasive medical devices in adult ICUs were included. Data were extracted on study characteristics, device type, microorganisms, biofilm detection methods, and clinical outcomes. Results were synthesized descriptively and presented through tables and an evidence map. **Results**: Sixteen studies met the inclusion criteria. Evidence was primarily focused on endotracheal tubes, vascular catheters, and urinary catheters. Biofilm formation was consistently documented across these devices, involving Gram-negative bacteria, Gram-positive cocci, and fungal pathogens. Detection methods were heterogeneous, with early reliance on electron microscopy and increasing use of quantitative phenotypic assays, while molecular approaches remained limited. The evidence map revealed a concentration of studies in vascular devices and significant methodological variability across studies. **Conclusions**: Biofilm formation on invasive medical devices in adult ICUs is a clinically significant and microbiologically complex phenomenon. The available evidence highlights key device categories and pathogen profiles but is limited by methodological heterogeneity and gaps in molecular characterization and translational research. Future studies should prioritize standardized, multimodal approaches and clinically oriented designs to improve prevention and management of device-associated biofilm infections in critical care settings.

## 1. Introduction

The clinical impact of medical device-associated biofilms in intensive care units (ICUs) is starkly reflected in available morbidity and mortality statistics. Healthcare-associated infections (HAIs) linked to devices constitute three of the most serious infectious syndromes in intensive care: ventilator-associated pneumonia (VAP), central venous catheter-associated bloodstream infection (CVC-BSI), and catheter-associated urinary tract infection (CAUTI). According to data from the Centers for Disease Control and Prevention (CDC), approximately 687,000 HAIs occur annually in the United States, of which about 72% are related to the use of invasive devices [1]. VAP, directly linked to biofilm formation in the endotracheal tube, affects between 9% and 27% of mechanically ventilated patients in ICUs, with attributable mortality rates ranging from 20% to 50% in high-risk populations [2,3]. CVC-BSI, whose pathogenesis involves biofilm-mediated colonization of the internal and external surface of the catheter, is associated with mortality rates of up to 25% and with an average prolongation of hospital stay of between 7 and 21 days, with costs exceeding USD 45,000 per episode in high-income countries [4,5].

CAUTI represents one of the most common HAIs in adult ICUs, accounting for up to 40% of all reported device-associated infection episodes. Although its direct mortality is generally lower than that observed for ventilator-associated pneumonia and catheter-related bloodstream infections, its contribution to secondary bacteremia and the selection of multidrug-resistant microorganisms remains clinically significant [6]. Beyond their direct clinical consequences, healthcare-associated infections associated with invasive medical devices impose a substantial burden on healthcare systems worldwide. Biofilm-associated infections frequently result in recurrent infections, prolonged antimicrobial treatment, increased healthcare resource utilization, higher healthcare costs, and greater complexity of clinical management, particularly when device removal or replacement becomes necessary [7,8]. This burden is especially relevant in ICUs, where critically ill patients are highly dependent on invasive devices and are frequently exposed to broad-spectrum antimicrobial therapy [9]. In low- and middle-income countries, limitations in infection prevention infrastructure, microbiological diagnostic capacity, surveillance systems, and antimicrobial stewardship programs may further increase the incidence and clinical impact of healthcare-associated infections, contributing to higher morbidity, mortality, and healthcare expenditures [10]. In addition, device-associated infections caused by biofilm-forming pathogens represent an important challenge for infection prevention and control programs because they contribute to antimicrobial resistance and treatment failure [11]. Given that biofilm formation is a central mechanism underlying microbial persistence and infection chronicity, understanding its role is essential for improving clinical outcomes and strengthening infection prevention strategies in critical care settings.

Globally, biofilms are estimated to be responsible for more than 80% of chronic and recurrent infections affecting hospitalized patients, and 65% of infections requiring medical attention have a biofilm component [12,13]. This epidemiological burden underscores the widespread clinical significance of biofilm-associated infections across healthcare settings. The high prevalence of device-associated infections highlights the importance of understanding the biological mechanisms that enable microbial persistence on invasive medical devices. It is estimated that between 60% and 70% of nosocomial infections are directly related to the use of invasive medical devices [9]. The pathogenesis of this phenomenon is based on the formation of biofilms, defined as highly organized microbial communities protected by a self-generated matrix of extracellular polymeric substances that include proteins, extracellular DNA, and polysaccharides [14].

Biofilm formation is a dynamic and sequential process that can be divided into four well-defined stages. The first corresponds to initial reversible adhesion, in which planktonic microorganisms approach an abiotic surface, such as the polymer of a catheter or the silicone of an endotracheal tube, through Van der Waals forces, electrostatic interactions, and appendages such as pili and flagella [15]. This initial association is still reversible and depends on the physicochemical properties of the device surface, as well as the hydrophobicity and adhesin molecules of the bacteria [16]. In a second stage, the microorganisms transition to irreversible adhesion, mediated by specific anchoring structures, such as the MSCRAMM (microbial surface components recognizing adhesive matrix molecules) surface adhesins of *Staphylococcus aureus*, which bind to host proteins deposited on the surface of the device, such as fibronectin, fibrinogen, and vitronectin, constituting the so-called conditioning layer [13,17]. Once stably attached, the cells begin to proliferate and communicate through molecular signals, giving rise to three-dimensional microcolonies that produce and secrete the extracellular polymeric substance (EPS) matrix, composed mainly of polysaccharides, proteins, extracellular DNA, and lipids [14]. This matrix confers structural cohesion to the microbial community and acts as a selective barrier against antimicrobial agents and the host’s immune response [18]. The third stage corresponds to the maturation of the biofilm: the three-dimensional architecture consolidates into microbial towers and mushrooms traversed by aqueous channels that function as a primitive circulatory system, facilitating the transport of nutrients and the elimination of metabolites [15,18]. In this phase, phenotypic differentiation within the biofilm generates subpopulations with different metabolic states, including persistent cells with low metabolic activity, whose intrinsic tolerance to antibiotics poses one of the greatest therapeutic challenges in the ICU environment [19]. Finally, the fourth stage of dispersal involves the active release of planktonic cells from the mature biofilm, enabling the spread of the infection to new niches or tissues of the patient, which constitutes a critical mechanism of persistence and recurrence in device-associated infections [16,20]. This structure not only enables stable microbial colonization of synthetic surfaces but also transforms invasive medical devices into persistent reservoirs that challenge conventional infection prevention and treatment strategies. Once established, biofilms promote long-term microbial survival and contribute to multidrug-resistant device-associated infections [7]. These biofilm-associated infections frequently complicate clinical management, prolong treatment requirements, and contribute to increased morbidity among hospitalized patients [8]. These challenges are further exacerbated by the prolonged use of invasive devices, the critical condition of patients, and the selective pressure exerted by broad-spectrum antimicrobial therapy in critical care settings [9]. Therefore, biofilm formation should be understood not only as a microbiological process but also as a clinically relevant mechanism contributing to persistent colonization, therapeutic failure, and adverse outcomes in critically ill patients.

The growing recognition of the clinical importance of biofilm-associated infections has stimulated the development of multiple approaches for their detection and characterization. Structural techniques such as scanning electron microscopy (SEM) and transmission electron microscopy (TEM) enable direct visualization of biofilm architecture on device surfaces and have historically served as reference methods for biofilm confirmation [15]. Quantitative phenotypic methods, including microtiter plate assays and crystal violet staining, are widely used because they provide standardized and relatively inexpensive assessments of biofilm-forming capacity [16]. More recently, molecular approaches such as polymerase chain reaction (PCR) and sequencing-based technologies have facilitated the detection of biofilm-associated genes, virulence factors, and complex microbial communities [11]. However, despite their scientific value, many of these techniques remain difficult to implement routinely in clinical microbiology laboratories because of technical complexity, cost, limited standardization, and challenges in distinguishing microbial colonization from clinically significant infection [7].

One of the most critical aspects of biofilms is their central role in the global antimicrobial resistance crisis. Bacteria embedded in them can exhibit antibiotic resistance 10 to 1000 times greater than that of their planktonic counterparts [21]. This phenomenon is explained by multiple mechanisms: the matrix of extracellular polymeric substances acts as a physical barrier that limits the penetration of antimicrobials; in addition, the metabolic heterogeneity within the biofilm favors the appearance of persistent subpopulations with low metabolic activity, less susceptible to drugs directed at active growth processes [14]. Furthermore, the biofilm microenvironment facilitates horizontal gene transfer, promoting the rapid spread of resistance determinants between different bacterial species [21]. Consequently, infections develop that are refractory to conventional treatment, frequently requiring removal of the medical device as an essential therapeutic measure [13]. In the specific case of the endotracheal tube, biofilm can form early and persistently, acting as a reservoir from which bacteria can migrate to the lower respiratory tract during mechanical ventilation, thus promoting the development of ventilator-associated pneumonia [3]. Similarly, the use of intravascular devices is associated with bloodstream infections that can reach mortality rates of up to 25% [4]. Given this scenario, current research has explored various preventive and therapeutic strategies, from non-adherent coatings to multimodal approaches aimed at biofilm matrix degradation and improving antimicrobial efficacy [22]. In this context, this scoping review aimed to map and characterize the available biomedical, microbiological, and clinical evidence on biofilm formation on invasive medical devices used in adult ICUs.

## 2. Materials and Methods

This scoping review was conducted in accordance with the PRISMA-ScR guidelines [23] (File S1). The methodological protocol was developed a priori by the research team and included the research question (PCC), eligibility criteria, search strategy, selection process, plan for graph development, and synthesis approach. This scoping review was registered in the Open Science Framework (OSF). The registration is publicly available at: https://doi.org/10.17605/OSF.IO/G25YU. The registration was completed to improve methodological transparency, reproducibility, and documentation of the review process.

### 2.1. Research Question (PCC Framework)

This scoping review was structured according to the Population–Concept–Context (PCC) framework, recommended for this type of review.

- Population: Microorganisms isolated from invasive medical devices used in adult ICUs.
- Concept: Formation and detection of microbial biofilms, including assessment methods, microbial composition, virulence factors, and associated antimicrobial resistance patterns.
- Context: Adult ICUs, considering primarily invasive and functionally invasive medical devices used in critical care.

Based on this framework, the guiding question for this review was: What is the scope, nature, and characteristics of the available biomedical, microbiological, and clinical evidence on biofilm formation on invasive medical devices used in adult ICUs?

### 2.2. Information Sources and Search Strategy

A specialized search was conducted in the electronic databases PubMed, Embase, Scopus, Web of Science, and ScienceDirect, from database inception to 23 December 2025. This was supplemented by a manual search of the reference lists of key and relevant articles. The search strategies were designed to be fully reproducible. The search strategy was developed according to the main concepts defined in the PCC framework (“Biofilms”, “Equipment and Supplies”, and “Intensive Care Units”). Controlled vocabulary terms from MeSH [https://www.ncbi.nlm.nih.gov/mesh/ (accessed on 23 December 2025)] and DeCS [https://decs.bvsalud.org/en/ (accessed on 23 December 2025)], together with corresponding Entry Terms and equivalent text-word terms, were used to maximize search sensitivity across databases with different indexing systems. The specific search terms and their database-specific adaptations are presented in Table 1. No language or publication date restrictions were applied. Reference lists of relevant articles and previous systematic reviews were also manually reviewed to identify additional eligible studies. Grey literature sources were excluded.

### 2.3. Eligibility Criteria

Eligibility criteria were defined a priori. Studies were included if they: (i) assessed biofilm formation on invasive medical devices used in adult ICUs; (ii) focused on primarily invasive or functionally invasive devices, such as vascular catheters, urinary catheters, and endotracheal tubes; (iii) reported microbiological isolation/identification and/or methods for biofilm detection, characterization, or quantification; and (iv) were primary studies. The following were excluded: (i) studies outside the context of adult ICUs, including pediatric or neonatal ICUs settings; (ii) studies without direct biofilm assessment on devices or device-derived samples; (iii) studies focused exclusively on environmental surfaces not directly related to invasive medical devices; and (iv) secondary studies, including narrative reviews, systematic reviews, meta-analyses, scoping reviews, letters to the editor, opinion articles, and conference abstracts.

### 2.4. Selection of Evidence Sources (Screening)

All retrieved references were exported to Mendeley (https://www.mendeley.com/) and subsequently to Rayyan (https://www.rayyan.ai/) for duplicate removal and screening. The selection process was carried out in two sequential phases: (i) title and abstract screening and (ii) full-text assessment. Each phase was performed independently by reviewers using the predefined eligibility criteria. Discrepancies were resolved by consensus and, when necessary, by consultation with an additional reviewer. Prior to formal screening, a pilot exercise was conducted using a sample of records to standardize the application of the eligibility criteria and refine the screening form. To ensure consistency with the final scope of this review, particular attention was given during full-text assessment to whether studies directly evaluated biofilm formation on invasive medical devices or device-derived samples used in adult ICUs.

### 2.5. Data Extraction Process

A standardized data-charting form was developed a priori and piloted to ensure consistency and completeness of data extraction. Data extraction was performed independently and in triplicate. Operational rules were defined for complex variables, such as biofilm definition, detection or quantification method, device categorization, microbiological outcomes, and clinical relevance. Data elements were defined according to the review objectives and the PCC framework. Extracted variables included bibliographic, methodological, microbiological, and clinical information relevant to biofilm formation on invasive medical devices used in adult ICUs.

Specifically, the following data were extracted:

- General study characteristics: author(s), year of publication, country, adult ICUs setting, study design, and sample size (when reported);
- Invasive medical device characteristics: type of invasive medical device evaluated, device category, classification as primary invasive or functionally invasive (when applicable), material (when reported), and unit of analysis, such as catheter tips, endotracheal tubes, urinary catheters, device surfaces, secretions, or aspirates;
- Microbiological characterization: microorganisms identified, microbial category (Gram-positive bacteria, Gram-negative bacteria, fungi, or polymicrobial communities), and methods of microbiological identification;
- Biofilm assessment: methods used for the detection, characterization, or quantification of biofilms, including electron microscopy, microtiter plate assays, cell culture plate assays, tube methods, culture-based approaches, and molecular techniques. The evidence was categorized according to the reported method and whether biofilm formation was directly demonstrated;
- Antimicrobial resistance and virulence factors: antimicrobial susceptibility profiles, multidrug resistance patterns, biofilm-associated genes, and virulence factors (when reported);
- Associated clinical outcomes: colonization, device-associated infection, ventilator-associated pneumonia, catheter-associated urinary tract infection, catheter-related bloodstream infection, outbreaks, microbiological persistence, or other clinically relevant outcomes.

Where necessary, classification decisions were made to standardize the categories of devices, methods, and microorganisms across all studies. These decisions were documented during the data extraction process to ensure transparency and reproducibility. A formal critical appraisal of methodological quality and risk of bias was not performed, consistent with the purpose of this exploratory review, which was to describe the scope, nature, and characteristics of the available evidence, rather than to estimate the effects of interventions or compare outcomes across studies.

### 2.6. Synthesis of Results

The results were synthesized descriptively, following the methodological approach of scoping reviews. Given the heterogeneity of study designs, biofilm assessment methods, device types, microorganisms, and reported outcomes, a meta-analysis was not performed.

The evidence was organized and presented using:

- General study characteristics, including author(s), year of publication, country, adult ICUs setting, study design, and sample size when reported;
- Descriptive tables, summarizing the characteristics of the included studies, device categories, biofilm detection methods, microorganisms identified, antimicrobial resistance patterns, virulence factors, and associated clinical outcomes, when reported;
- A structured narrative synthesis, grouping the results according to device type, biofilm detection or characterization method, predominant microbial profiles, and clinically relevant outcomes, including device-associated infection, colonization, and microbiological persistence;
- An evidence map, designed to visualize the distribution of studies according to device category and biofilm detection method, allowing identification of evidence concentrations and research gaps.

The synthesis aimed to identify recurring patterns, concentrations of evidence, and gaps in the available biomedical, microbiological, and clinical literature on biofilm formation on invasive medical devices used in adult ICUs.

## 3. Results

### 3.1. Selection of Sources of Evidence

The search strategy conducted on 23 December 2025 identified a total of 929 records distributed as follows: PubMed (n = 149), Scopus (n = 241), Web of Science (n = 181), ScienceDirect (n = 19), and Embase (n = 339). After removing 404 duplicate records, 525 unique records were screened by title and abstract. During title and abstract screening, 504 records were excluded because they did not satisfy the predefined eligibility criteria. The principal reasons for exclusion were that the studies were conducted outside adult ICU settings, did not directly evaluate biofilm formation on invasive or functionally invasive medical devices or device-derived samples, focused exclusively on environmental surfaces unrelated to invasive medical devices, or corresponded to non-primary publications. A total of 21 reports were sought for retrieval, of which four could not be retrieved in full text. The remaining 17 full-text articles were assessed for eligibility, and one article was excluded because it did not directly assess biofilm formation. Finally, 16 studies met the established eligibility criteria and were included in this scoping review. The complete selection process is presented in the PRISMA-ScR flow diagram (Figure 1).

### 3.2. Characteristics of Included Studies

A total of 16 studies published between 1986 and 2024 met the inclusion criteria and were included in the final analysis [24,25,26,27,28,29,30,31,32,33,34,35,36,37,38,39]. The temporal distribution of the literature demonstrates a progressive expansion of research on biofilm formation associated with invasive medical devices used in adult ICUs. The main characteristics of the included studies are summarized in Table 2.

Geographically, the included studies originated from several regions worldwide, including the United States [34,39], Canada [27], the United Kingdom [37], Ireland [38], Austria [36], France/Algeria [26], Algeria [30], Iran [28], India [24,25,35], Tunisia [31], Indonesia [33], Sri Lanka [29], and Egypt [32]. This geographic diversity highlights the global relevance of device-associated biofilm formation in hospital environments.

Regarding study design, the included literature comprised several methodological approaches. The majority of studies were observational microbiological or clinical investigations analyzing microbial colonization and biofilm formation on invasive medical devices or clinical isolates [24,25,27,28,29,30,32,33,34,35,36,37,38,39]. Among these, several studies employed prospective or cross-sectional clinical designs, particularly when investigating device-associated infections in adult ICUs [29,32,33,35]. Other studies adopted laboratory-based observational approaches, focusing on phenotypic or molecular characterization of biofilm-forming isolates [28,38]. In addition, a smaller group of investigations employed experimental microbiological designs aimed at characterizing biofilm formation under controlled conditions or using catheter models [26,31]. These experimental studies provided mechanistic insights into biofilm development and virulence traits associated with device-associated infections.

Across the included studies, three principal categories of invasive medical devices were investigated. Several studies focused on endotracheal tubes, particularly in relation to ventilator-associated pneumonia [33,34,37,39]. Another important group examined vascular or central venous catheters, which are commonly associated with bloodstream infections [24,27,29,30,35]. In addition, urinary catheters were evaluated in studies investigating catheter-associated urinary tract infections [25,26,32]. Some investigations also analyzed microbial isolates linked to device-associated infections in ICUs settings [28,38], while others explored fungal biofilms associated with catheter use [31,36].

The included studies employed diverse methodologies to evaluate biofilm formation. Early studies relied primarily on electron microscopy techniques, including scanning electron microscopy (SEM) and transmission electron microscopy (TEM), to directly visualize biofilm structures on device surfaces [26,27,34,36,37,39]. More recent studies increasingly incorporated phenotypic quantitative assays, particularly the microtiter plate method, tissue culture plate assay, or crystal violet staining, which allowed standardized evaluation of biofilm formation [24,25,28,29,32,33,35,38]. In addition, several investigations employed molecular techniques, including PCR detection of biofilm-associated genes such as the *ica* operon [28,32,38] or microbial community analysis based on 16S rRNA sequencing [39].

The microorganisms reported across the included studies belonged mainly to three groups. Gram-negative bacteria were commonly identified in respiratory and urinary devices, including *Pseudomonas aeruginosa*, *Acinetobacter baumannii*, *Klebsiella* spp., *Escherichia coli*, and *Proteus mirabilis* [25,26,32,33,34,39]. Gram-positive bacteria, particularly *Staphylococcus aureus*, *Staphylococcus epidermidis*, and other coagulase-negative *staphylococci*, were frequently reported in catheter-associated infections [24,27,28,29,35,38]. In addition, fungal biofilms, especially involving *Candida* species and *Yarrowia lipolytica*, were described in several studies focusing on catheter-associated infections [30,31,36]. Overall, the evidence indicates that biofilm formation on invasive medical devices used in adult ICUs represents a key factor contributing to healthcare-associated infections, involving a broad spectrum of bacterial and fungal pathogens.

### 3.3. Invasive Medical Devices Associated with Biofilm Formation

Across the included studies, biofilm formation was predominantly investigated in invasive medical devices used in adult intensive care settings, particularly those with prolonged contact with biological fluids and tissues. The most frequently studied devices were endotracheal tubes, vascular or central venous catheters, and urinary catheters (Table 3).

Studies focusing on endotracheal tubes consistently demonstrated the presence of biofilm associated with respiratory pathogens and ventilator-associated pneumonia, as reported by Sottile et al. [34] and Adair et al. [37], with additional molecular characterization provided by Perkins et al. [39] and quantitative assessment by Dewi et al. [33]. These findings highlight the role of biofilm formation as an early event in microbial colonization of the lower respiratory tract. Similarly, vascular and central venous catheters represented the most extensively studied device category. Evidence from Passerini et al. [27], Seddiki et al. [30], Kaur et al. [35], Medis et al. [29], Singh et al. [24] and Paulitsch et al. [36] reported microbial colonization and biofilm formation on catheter surfaces, frequently associated with bloodstream infections, fungal colonization, or persistent device-associated infection. In addition, urinary catheters were investigated in the context of catheter-associated urinary tract infections, where biofilm formation was consistently documented [25,26,32]. Other studies focused on ICU isolates or experimental models of device-associated infection, further supporting the relevance of biofilm formation across different clinical contexts [28,31,38]. Overall, the distribution of devices indicates that research on biofilm formation in adult ICUs is primarily concentrated on devices that breach or bypass anatomical barriers, facilitating microbial adherence and persistence.

Across the included studies, biofilm formation was frequently associated with clinically relevant outcomes. Biofilms identified on endotracheal tubes were associated with ventilator-associated pneumonia, as reported by Sottile et al. [34], who demonstrated bacterial adhesion and biofilm development on endotracheal tube surfaces, and by Adair et al. [37], who highlighted the clinical implications of endotracheal tube biofilms in the pathogenesis of ventilator-associated pneumonia. Subsequent studies by Perkins et al. [39] and Dewi et al. [33] further supported the role of endotracheal tube-associated biofilms as reservoirs for respiratory pathogens involved in ventilator-associated pneumonia. Similarly, biofilm formation on urinary catheters was consistently linked to catheter-associated urinary tract infections. Djeribi et al. [26] demonstrated biofilm development on urinary catheter surfaces by clinically relevant Gram-negative pathogens. Patel et al. [25] reported biofilm-producing microorganisms among pathogens isolated from catheter-associated urinary tract infections in ICU patients, whereas Mohamed et al. [32] identified biofilm-forming *Escherichia coli* isolates carrying virulence-associated genes in urinary catheter-associated infections.

In vascular and central venous catheters, biofilm formation was commonly associated with catheter colonization and bloodstream infection. Passerini et al. [27] documented biofilm formation on indwelling vascular catheters, while Kaur et al. [35] investigated venous catheter-associated bloodstream infections and identified biofilm-producing pathogens among the recovered isolates. More recently, Medis et al. [29] reported biofilm-forming coagulase-negative staphylococci recovered from central venous catheters of ICU patients, and Singh et al. [24] described microbial colonization of indwelling vascular catheters associated with biofilm formation. Collectively, these findings reinforce the clinical significance of biofilm formation as a contributing factor in device-associated infections in adult intensive care units.

### 3.4. Microorganisms Associated with Device-Related Biofilms

The microorganisms identified across the included studies were diverse and can be broadly categorized into Gram-negative bacteria, Gram-positive bacteria, and fungi (Table 3). Gram-negative bacteria were frequently identified in endotracheal and urinary devices, including *Pseudomonas aeruginosa*, *Acinetobacter baumannii*, *Klebsiella* spp., *Escherichia coli*, and *Proteus mirabilis* [25,26,32,33,34,39]. Gram-positive bacteria, particularly *Staphylococcus aureus*, *Staphylococcus epidermidis*, and other coagulase-negative staphylococci, were predominantly reported in studies involving vascular catheter-related infections or ICU isolates associated with device-related biofilms [24,27,28,29,35,38]. In addition, fungal biofilms, mainly involving *Candida* species and *Yarrowia lipolytica*, were reported in studies focused on catheter-associated infections or catheter-related experimental models [30,31,36]. Overall, the evidence indicates that biofilm-associated infections in adult ICUs involve bacterial and fungal pathogens, with distribution patterns influenced by device type and clinical context.

### 3.5. Biofilm Detection Methods

A wide range of methodologies was used to detect and characterize biofilm formation across the included studies (Table 4; Figure 2). Early studies predominantly relied on electron microscopy techniques, including scanning electron microscopy (SEM) and transmission electron microscopy (TEM), which allowed direct visualization of biofilm structures on device surfaces [26,27,30,34,36,37].

More recent investigations increasingly employed phenotypic quantitative assays, particularly microtiter plate-based methods, which enable standardized measurement of biofilm formation [25,28,29,31,33,35,38]. Among the phenotypic approaches, several studies employed microtiter plate-based assays using crystal violet staining to estimate total biofilm biomass, including Djeribi et al. [26], Patel et al. [25], Mirzaee et al. [28], Mohamed et al. [32], Dewi et al. [33], and Fitzpatrick et al. [38]. Kaur et al. [35] used the tissue culture plate method to classify isolates according to their biofilm-forming capacity, whereas Singh et al. [24] combined conventional culture techniques with the tube method for qualitative biofilm assessment. Medis et al. [29] incorporated phenotypic biofilm assays together with antimicrobial susceptibility testing of catheter-associated isolates. These methods primarily evaluated biofilm biomass and adherence capacity, whereas direct structural characterization of the extracellular matrix was mainly achieved through electron microscopy techniques. In addition, several studies incorporated molecular approaches, including PCR detection of biofilm-associated genes such as the ica operon [28,32,38], as well as microbial community profiling based on 16S rRNA sequencing [39]. Other complementary approaches included culture-based methods and phenotypic assays, particularly in studies evaluating clinical isolates [24,35]. Overall, these findings indicate a methodological evolution from structural visualization to quantitative and molecular characterization, although substantial heterogeneity persists across studies.

A limited number of studies explored the relationship between biofilm formation and antimicrobial resistance. Patel et al. [25] reported that biofilm-producing pathogens isolated from catheter-associated urinary tract infections frequently exhibited multidrug-resistant phenotypes. Similarly, Medis et al. [29] identified biofilm-forming coagulase-negative staphylococci recovered from central venous catheters that displayed clinically relevant antimicrobial resistance patterns. In addition, Mohamed et al. [32] evaluated virulence-associated genes in biofilm-forming Escherichia coli isolates obtained from urinary catheter-associated infections, highlighting the coexistence of biofilm production and pathogenicity-related determinants.

## 4. Discussion

This scoping review mapped the available biomedical, microbiological, and clinical evidence on biofilm formation on invasive medical devices used in adult ICUs. The findings confirm that biofilm formation is a central mechanism underlying device-associated infections, particularly in endotracheal tubes, vascular catheters, and urinary catheters. These devices share key characteristics, including prolonged contact with host tissues, exposure to biological fluids, and disruption or bypassing of anatomical barriers, which favor microbial adhesion, biofilm maturation, and persistence. This interpretation is consistent with current evidence describing biofilms as major determinants of chronic device-associated infection and antimicrobial tolerance [17,21,40].

A major finding of this review was the predominance of vascular and central venous catheter-related studies. Early structural evidence of biofilm formation on intravascular catheters was provided by Passerini et al. [27], while subsequent studies confirmed microbial colonization and persistence in catheter-associated clinical contexts [24,29,30,35,36]. This is consistent with contemporary evidence showing that catheter-related bloodstream infections and central line-associated bloodstream infections remain major targets for infection prevention in acute-care hospitals [41,42]. In this context, biofilm formation is clinically relevant because it contributes to sustained microbial colonization, antimicrobial tolerance, and the need for catheter removal when infection cannot be controlled [21,43].

Although no studies specifically evaluating cardiac implantable electronic devices (CIEDs) met the eligibility criteria for inclusion in this scoping review, these devices represent a clinically important category of invasive medical devices affected by biofilm-related infections. Biofilm-mediated colonization of pacemakers, implantable cardioverter-defibrillators, cardiac resynchronization therapy systems, and transvenous leads contributes to chronic infection, bacteremia, infective endocarditis, and increased healthcare utilization, frequently requiring complete device and lead extraction because antimicrobial therapy alone is often insufficient [44,45]. Recent preventive strategies, including individualized risk assessment, peri-procedural infection prevention measures, and antimicrobial prophylaxis, have shown promise in reducing CIED-associated infections [44,46]. Although outside the scope of the present review, these findings reinforce the broader clinical relevance of biofilm-associated infections across invasive medical devices and identify CIEDs as an important area for future biofilm-focused research.

Endotracheal tubes represented another critical device category. The included studies consistently demonstrated biofilm formation on these devices [33,34,37,39], supporting their role as reservoirs for respiratory pathogens associated with ventilator-associated pneumonia (VAP). Structural evidence from Sottile et al. [34] and Adair et al. [37], combined with molecular characterization by Perkins et al. [39] and quantitative assessment by Dewi et al. [33], highlights the complexity of airway device-associated biofilms. This is consistent with contemporary clinical evidence indicating that VAP remains one of the most important infectious complications in mechanically ventilated adults [2].

Urinary catheters were also consistently associated with biofilm formation, particularly in catheter-associated urinary tract infections [25,26,32]. These studies identified Gram-negative bacteria such as *Escherichia coli*, *Klebsiella* spp., *Pseudomonas aeruginosa*, and *Acinetobacter baumannii* as relevant pathogens. This aligns with established evidence indicating that biofilm formation contributes to persistence, recurrence, and antimicrobial tolerance in catheter-associated urinary tract infections [6,47]. In addition, an experimental study on urinary catheter polymicrobial biofilms showed that microbial interactions can influence biofilm structure and behavior, supporting the relevance of studying catheter biofilms as complex microbial communities rather than single-pathogen events [48].

From a microbiological perspective, the included evidence confirms the involvement of Gram-negative bacteria, Gram-positive bacteria, and fungi in device-associated biofilms. Gram-negative pathogens were commonly identified in respiratory and urinary devices [25,26,32,33,34,39], whereas Gram-positive organisms, particularly *Staphylococcus aureus*, *Staphylococcus epidermidis*, and coagulase-negative staphylococci, were frequently reported in catheter-related infections or ICU isolates [24,27,28,29,35,38]. This pattern is biologically plausible because staphylococci have well-described adhesion and biofilm-forming mechanisms on biomaterials, while Gram-negative bacteria are frequently associated with severe ICU infections and antimicrobial resistance [17,21].

A particularly relevant finding was the presence of fungal biofilms, mainly involving *Candida* spp. and *Yarrowia lipolytica* [30,31,36]. Although less frequently represented than bacterial biofilms, fungal biofilms are clinically significant because they are associated with catheter-related candidemia, antifungal tolerance, and difficult eradication. Current clinical evidence confirms that Candida biofilms remain a major challenge in device-associated infections and often require combined antifungal and device-management strategies [49,50].

One of the most important contributions of this review is the identification of methodological heterogeneity in biofilm detection. Early studies relied mainly on structural techniques such as scanning or transmission electron microscopy [26,27,30,34,36,37], which allow direct visualization of biofilm architecture on device surfaces but are difficult to implement in routine clinical workflows. Electron microscopy techniques provide high-resolution structural information and remain the reference standard for direct visualization of biofilm architecture [15,40]. However, they require specialized equipment, trained personnel, extensive sample preparation, and are not readily applicable to routine clinical microbiology workflows [40,43]. In contrast, microtiter plate and tissue culture plate assays are inexpensive, reproducible, and suitable for high-throughput screening of biofilm-forming isolates [11,16]. Nevertheless, these methods primarily quantify biofilm biomass or adherence capacity and may not accurately reproduce the complex biological conditions present on medical devices in vivo [40,43]. Molecular approaches offer the additional advantage of detecting biofilm-associated genes, virulence determinants, polymicrobial communities, and non-culturable microorganisms [21,40]. However, they cannot distinguish viable microorganisms from residual microbial DNA and therefore require careful interpretation within the clinical context [43]. More recent studies incorporated phenotypic quantitative assays, particularly microtiter plate-based methods [25,28,29,31,32,33,35,38]. These assays improve comparability across isolates but do not fully reproduce in vivo ICU conditions, including flow dynamics, conditioning layers, polymicrobial interactions, immune factors, and antimicrobial exposure [40,43]. Molecular methods were comparatively underrepresented, with only a few studies employing PCR or sequencing-based approaches [28,32,39]. This is a relevant gap because molecular techniques can support detection of polymicrobial communities, biofilm-associated genes, virulence markers, and non-culturable organisms. However, molecular detection also requires careful interpretation, since microbial DNA does not necessarily indicate viable biofilm or active infection. Therefore, future studies should prioritize integrated approaches combining structural visualization, phenotypic quantification, culture, antimicrobial susceptibility testing, and molecular characterization [40,43].

The evidence map further revealed important gaps in the available literature. First, most studies were observational or descriptive, with limited interventional research evaluating strategies to prevent or disrupt biofilms. Second, antimicrobial resistance and virulence factors were inconsistently reported despite their relevance for clinical decision-making. Third, evidence was concentrated on a limited number of device categories, while other ICU equipment remains underexplored. Fourth, only a minority of studies integrated molecular tools with phenotypic or structural methods. These gaps are particularly relevant given the growing interest in antibiofilm strategies, antimicrobial coatings, biofilm-disrupting compounds, and multimodal approaches for biofilm eradication [21,22,51]. Additionally, the material composition and surface characteristics of medical devices were rarely reported, limiting assessment of their potential influence on microbial adhesion, biofilm maturation, and infection risk. Future studies should systematically document device materials and surface properties to facilitate comparisons across studies and support the development of antibiofilm device technologies.

From a translational perspective, the findings suggest that future research should move beyond documenting biofilm presence and toward clinically actionable models. Priority areas include standardized sampling from device surfaces, harmonized biofilm quantification protocols, biofilm-specific antimicrobial susceptibility testing, and evaluation of preventive strategies in clinically relevant device models. For vascular catheters, current prevention guidance emphasizes bundles and evidence-based Central Line-Associated Bloodstream Infection (CLABSI) prevention practices, but future work should better integrate microbiological biofilm endpoints into prevention studies [42]. For urinary catheters and airway devices, future studies should incorporate longitudinal sampling to evaluate biofilm dynamics over time and their relationship with clinical infection [2,48]. Beyond their microbiological relevance, the findings of this review highlight the important clinical consequences of biofilm formation on invasive medical devices used in adult intensive care units. The included studies consistently associated biofilm formation with device-associated infections, particularly ventilator-associated pneumonia, catheter-associated urinary tract infection, and catheter-related bloodstream infection, demonstrating that biofilms act as persistent reservoirs for microbial colonization and infection [25,34,35,37]. These observations are consistent with previous evidence indicating that biofilm-associated microorganisms exhibit enhanced tolerance to antimicrobial therapy due to the protective extracellular polymeric matrix, reduced metabolic activity of persister cells, and impaired antimicrobial penetration, thereby promoting persistent infection, recurrent colonization, and treatment failure [21,40,43]. Consequently, biofilm-associated infections frequently require prolonged antimicrobial therapy, removal or replacement of the infected device, and are associated with longer hospital stays, increased healthcare costs, and greater morbidity among critically ill patients [42,47]. Collectively, these findings reinforce the importance of implementing preventive strategies aimed at minimizing biofilm formation on invasive medical devices, while integrating infection prevention, antimicrobial stewardship, and appropriate device management as complementary measures to reduce the burden of device-associated infections in intensive care settings [42,44,45].

The findings of this review have important implications for infection control, antimicrobial stewardship, and device management in adult intensive care units. Clinically, biofilm involvement should be suspected in patients with persistent or recurrent device-associated infections, particularly when microbiological eradication is not achieved despite apparently appropriate antimicrobial therapy, when cultures remain repeatedly positive, or when infection recurs shortly after treatment discontinuation [43]. These scenarios are especially relevant in patients carrying long-term vascular catheters, urinary catheters, endotracheal tubes, or other invasive medical devices.

The reduced effectiveness of antimicrobial therapy in biofilm-associated infections is explained by multiple mechanisms, including restricted antimicrobial penetration through the extracellular polymeric matrix, altered microbial metabolic activity, the presence of persister cells, and enhanced horizontal transfer of antimicrobial resistance determinants [40]. Consequently, biofilm-associated infections frequently require prolonged antimicrobial exposure and may contribute to treatment failure and the emergence of multidrug-resistant organisms [7]. In many clinical scenarios, particularly catheter-related bloodstream infections and established device-associated infections, device removal or replacement becomes an essential component of source control because eradication of mature biofilms is often difficult to achieve with antimicrobial therapy alone [43].

Beyond their microbiological relevance, biofilm-associated infections have important consequences for patient outcomes. Persistent biofilm colonization has been associated with prolonged hospitalization, increased healthcare utilization, recurrent infectious episodes, greater antimicrobial exposure, and a higher risk of severe complications such as bacteremia and sepsis [7,43]. In critically ill patients, these factors may contribute to delayed clinical recovery, treatment failure, and increased morbidity, highlighting the importance of early recognition and effective management of biofilm-associated device infections.

From a diagnostic perspective, current ICU microbiology workflows remain largely dependent on conventional culture-based approaches. However, recent evidence suggests that combining traditional microbiological methods with biofilm-oriented approaches, including sonication, quantitative phenotypic assays, molecular detection of biofilm-associated genes, and sequencing-based microbial profiling, may improve the characterization of device-associated infections and facilitate more individualized therapeutic decision-making [52]. In parallel, increasing attention has been directed toward biofilm-specific antimicrobial susceptibility testing, as conventional planktonic susceptibility assays may not adequately reflect the antimicrobial tolerance observed in mature biofilms, highlighting an important area for future clinical microbiology implementation [53].

Preventive strategies should also be adapted according to device type. For vascular catheters, evidence-based insertion and maintenance bundles remain the cornerstone of CLABSI prevention [42]. For urinary catheters, minimizing unnecessary catheterization and reducing catheter dwell time continue to represent the most effective preventive measures [47]. For endotracheal tubes, interventions targeting ventilator-associated pneumonia prevention, including optimized airway management and secretion control, remain essential [2].

In addition, recent evidence from cardiac implantable electronic devices highlights the importance of individualized risk assessment for identifying patients at increased risk of device-associated infection and guiding preventive strategies [44]. Optimized antimicrobial prophylaxis and treatment protocols have also been emphasized as key components in reducing the incidence and improving the management of device-associated infections [45]. Collectively, these observations reinforce the need for multidisciplinary approaches integrating infection control, antimicrobial stewardship, microbiology, and device management to reduce the burden of biofilm-associated infections in critically ill patients.

This review has several strengths, including adherence to PRISMA-ScR guidelines, use of a PCC framework, inclusion of multiple databases, and synthesis through structured tables and an evidence map [23]. However, limitations should be acknowledged. The included studies were heterogeneous in design, device type, biofilm detection method, and reporting of clinical outcomes, which limits direct comparability. A formal critical appraisal was not performed, consistent with the objective of this scoping review, which was to map the extent, nature, and characteristics of the evidence rather than estimate intervention effects. Additionally, the focus on studies with direct biofilm assessment strengthened internal coherence but may have excluded broader evidence on microbial contamination of ICU environments. In addition, information regarding the material composition of invasive medical devices was inconsistently reported across studies, preventing assessment of its potential influence on microbial adhesion and biofilm formation.

Overall, this scoping review shows that biofilm formation on invasive medical devices used in adult ICUs is a clinically relevant and microbiologically complex phenomenon. The available evidence supports the importance of endotracheal tubes, vascular catheters, and urinary catheters as major substrates for biofilm formation. Nevertheless, methodological heterogeneity, limited use of molecular methods, and inconsistent reporting of antimicrobial resistance and virulence factors restrict comparability across studies. Future investigations should prioritize standardized, multimodal, and clinically oriented approaches to better characterize biofilm dynamics and support preventive and therapeutic strategies for device-associated infections in critical care.

## 5. Conclusions

This scoping review provides the first comprehensive synthesis of the available biomedical, microbiological, and clinical evidence on biofilm formation on invasive medical devices used in adult ICUs. The findings confirm that biofilms are a central mechanism underlying device-associated infections, particularly in endotracheal tubes, vascular catheters, and urinary catheters, where prolonged exposure to biological fluids and disruption of anatomical barriers facilitate microbial adhesion, persistence, and antimicrobial tolerance.

The evidence highlights a consistent involvement of Gram-negative bacteria, Gram-positive cocci, and fungal pathogens, reflecting the microbiological complexity of device-associated biofilms in ICU settings. Despite this, the available literature remains methodologically heterogeneous, with a predominance of structural and phenotypic approaches and a limited integration of molecular techniques capable of capturing polymicrobial communities and resistance mechanisms.

Importantly, this review identifies critical gaps, including the lack of standardized methodologies, limited interventional research, inconsistent reporting of antimicrobial resistance and virulence factors, and underrepresentation of certain device categories. These limitations restrict comparability across studies and hinder the translation of findings into clinical practice.

From a clinical and translational perspective, future research should prioritize multimodal and standardized approaches for biofilm detection and characterization, incorporate biofilm-specific antimicrobial susceptibility testing, and evaluate preventive and therapeutic strategies in clinically relevant device models. Strengthening the integration between microbiological evidence and clinical outcomes will be essential to improve the management of device-associated infections in critically ill patients. Overall, biofilm formation on invasive medical devices in adult ICUs represents a complex and clinically significant challenge that requires coordinated advances in microbiology, biomaterials, and clinical research to develop effective prevention and treatment strategies. Ultimately, reducing biofilm formation on invasive medical devices should be considered a priority for improving infection prevention, antimicrobial stewardship, and patient outcomes in adult intensive care units.

## Figures and Tables

**Figure 1:**
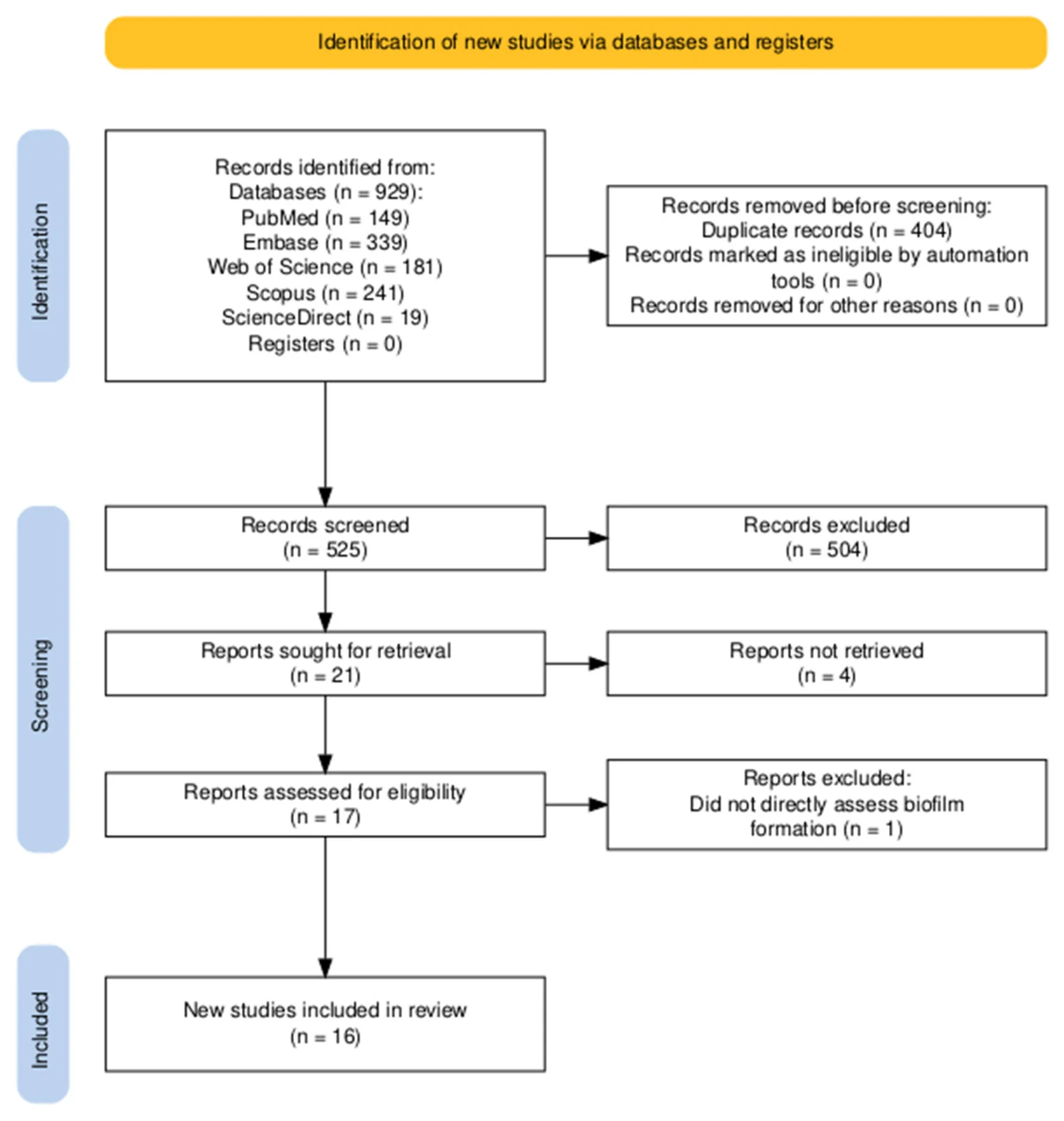
PRISMA-ScR flow diagram of the study selection process. The diagram illustrates the identification, screening, retrieval, eligibility assessment, and inclusion of studies in this scoping review. A total of 929 records were identified through electronic database searching. After removal of 404 duplicate records, 525 records underwent title and abstract screening, of which 504 were excluded according to the predefined eligibility criteria. Twenty-one reports were sought for retrieval; four could not be retrieved, and the remaining 17 full-text articles were assessed for eligibility using the predefined PCC-based eligibility criteria. One full-text article was excluded because it did not directly assess biofilm formation, resulting in a final sample of 16 studies included in this scoping review. Flowchart created in: https://estech.shinyapps.io/prisma_flowdiagram/ (accessed on 16 March 2026).

**Figure 2:**
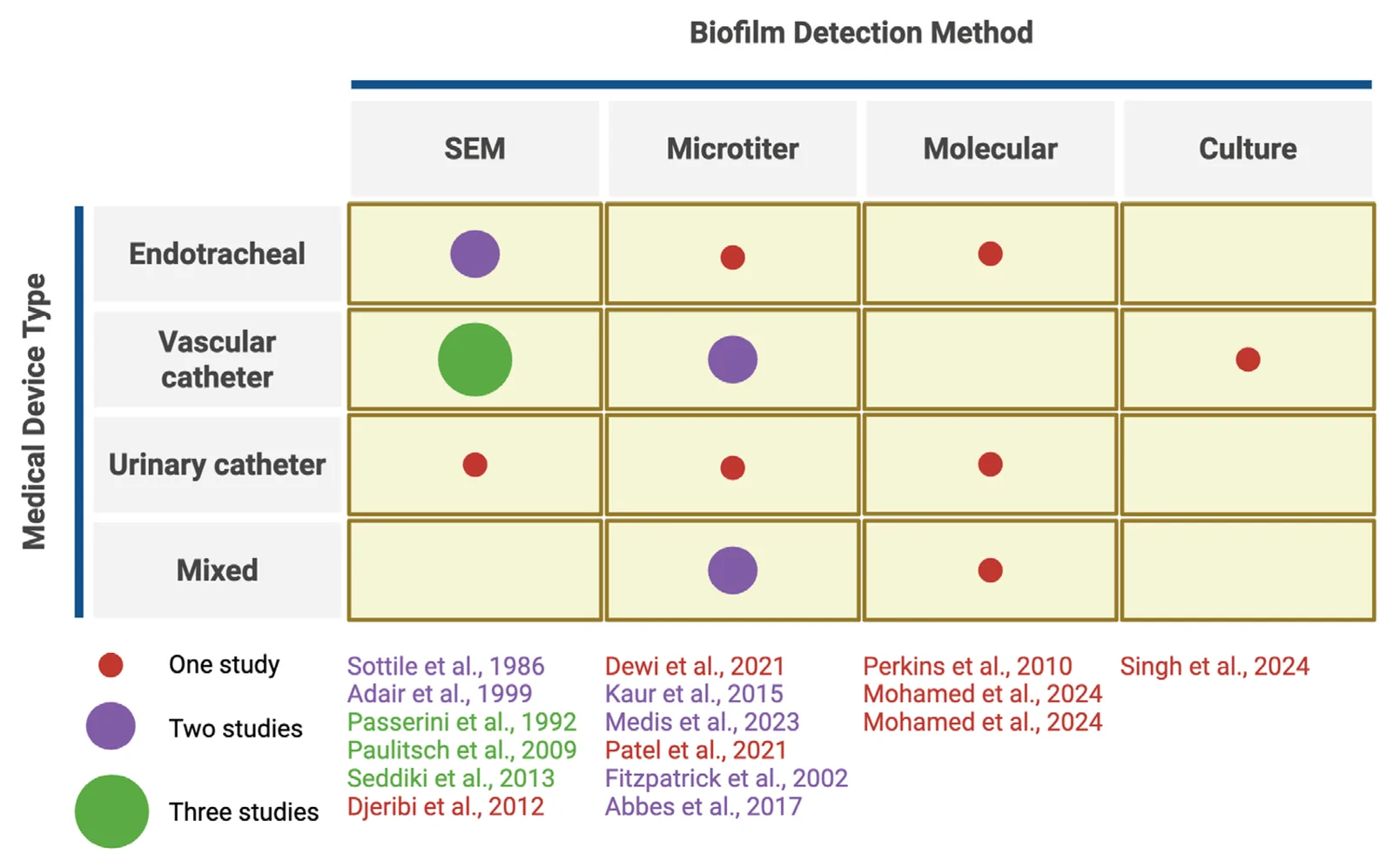
Evidence map of included studies according to medical device type and biofilm detection method. Bubble size represents the number of studies per category. The map shows a higher concentration of evidence for vascular catheters, particularly using SEM/TEM and microtiter plate assays. Endotracheal tube studies are primarily associated with structural, quantitative, and molecular approaches, while urinary catheter studies are distributed across SEM/TEM, microtiter, and molecular methods. Mixed or ICUs isolate/model studies are predominantly evaluated using microtiter and molecular techniques. The distribution highlights methodological heterogeneity and evidence gaps in molecular approaches for certain device types. Created in BioRender. Carrillo, J. (2026) https://BioRender.com/o1nle1f.

**Table 1:** Searching for strategy in electronic databases.

Database	Strategy	Results
PubMed	S1: “Biofilms” OR “Biofilm” S2: “Apparatus and Instruments” OR “Device” OR “Device, Medical” OR “Devices” OR “Devices, Medical” OR “Equipment” OR “Equipment and Supplies” OR “Instruments and Apparatus” OR “Inventories” OR “Inventory” OR “Medical Device” OR “Medical Devices” OR “Supplies” OR “Supplies and Equipment” S3: “Care Unit, Intensive” OR “Care Units, Intensive” OR “ICU Intensive Care Units” OR “Intensive Care Unit” OR “Intensive Care Units” OR “Unit, Intensive Care” OR “Units, Intensive Care” FS: (S1) AND (S2) AND (S3)	149
Scopus	S1: TITLE-ABS-KEY (“Biofilms” OR “Biofilm”) S2: TITLE-ABS-KEY (“Apparatus and Instruments” OR “Device” OR “Device, Medical” OR “Devices” OR “Devices, Medical” OR “Equipment” OR “Equipment and Supplies” OR “Instruments and Apparatus” OR “Inventories” OR “Inventory” OR “Medical Device” OR “Medical Devices” OR “Supplies” OR “Supplies and Equipment”) S3: TITLE-ABS-KEY (“Care Unit, Intensive” OR “Care Units, Intensive” OR “ICU Intensive Care Units” OR “Intensive Care Unit” OR “Intensive Care Units” OR “Unit, Intensive Care” OR “Units, Intensive Care”) FS: (S1) AND (S2) AND (S3)	241
Web of Science	S1: “Biofilms” OR “Biofilm” S2: “Apparatus and Instruments” OR “Device” OR “Device, Medical” OR “Devices” OR “Devices, Medical” OR “Equipment” OR “Equipment and Supplies” OR “Instruments and Apparatus” OR “Inventories” OR “Inventory” OR “Medical Device” OR “Medical Devices” OR “Supplies” OR “Supplies and Equipment” S3: “Care Unit, Intensive” OR “Care Units, Intensive” OR “ICU Intensive Care Units” OR “Intensive Care Unit” OR “Intensive Care Units” OR “Unit, Intensive Care” OR “Units, Intensive Care” FS: (S1) AND (S2) AND (S3)	181
Science Direct	S1: “Biofilms” OR “Biofilm” S2: “Apparatus and Instruments” OR “Device” OR “Device, Medical” OR “Devices” OR “Devices, Medical” OR “Equipment” OR “Equipment and Supplies” OR “Instruments and Apparatus” OR “Inventories” S3: “Care Unit, Intensive” OR “Care Units, Intensive” OR “ICU Intensive Care Units” OR “Intensive Care Unit” OR “Intensive Care Units” OR “Unit, Intensive Care” OR “Units, Intensive Care” FS: (S1) AND (S2) AND (S3)	19
Embase	S1: ‘bio-film’/exp OR ‘bio-film’ OR ‘bio-films’/exp OR ‘bio-films’ OR ‘biofilms’/exp OR ‘biofilms’ OR ‘biofilm’/exp OR ‘biofilm’ S2: ‘apparatus’ OR ‘apparatus, equipment and supplies’ OR ‘device’ OR ‘equipment’ OR ‘equipment and supplies’ OR ‘instrument’ OR ‘instrument, device’ OR ‘instrumentation’ OR ‘devices’ S3: ‘close attention unit’ OR ‘combined medical and surgical ICU’ OR ‘combined surgical and medical ICU’ OR ‘critical care unit’ OR ‘general ICU’ OR ‘GICU’ OR ‘GICUs’ OR ‘ICU`s’ OR ‘intensive care department’ OR ‘intensive care units’ OR ‘intensive therapy unit’ OR ‘intensive treatment unit’ OR ‘medical-surgery ICU’ OR ‘medical/surgical ICU’ OR ‘medical/surgical ICUs’ OR ‘medico-surgical ICU’ OR ‘mixed medical and surgical ICU’ OR ‘mixed surgical and medical ICU’ OR ‘respiratory care unit’ OR ‘respiratory care units’ OR ‘special care unit’ OR ‘surgery/medical ICU’ OR ‘surgical-medical ICUs’ OR ‘surgical/medical ICU’ OR ‘unit, intensive care’ OR ‘intensive care unit’ FS: (S1) AND (S2) AND (S3)	339
Total		929

S = strategy. FS = Final Strategy. OR/AND: Boolean Operators.

**Table 2:** General characteristics of the included studies.

Authors	Year	Country	Study Design	Medical Device Sample Source	Biofilm Detection Method	Main Microorganisms Reported
Sottile et al. [34]	1986	USA	Observational microbiological study	Endotracheal tubes	Scanning and transmission electron microscopy (SEM/TEM)	*Staphylococcus aureus*, *Pseudomonas* *aeruginosa*, *Proteus mirabilis*
Passerini et al. [27]	1992	Canada	Observational laboratory study	Indwelling vascular catheters	SEM and TEM microscopy	Mixed bacterial biofilms including staphylococci and Gram-negative bacilli
Adair et al. [37]	1999	United Kingdom	Observational clinical study	Endotracheal tubes	SEM and microbial culture	*Staphylococcus aureus*, *Enterobacteriaceae*, *Pseudomonas* spp., *Candida* spp.
Fitzpatrick et al. [38]	2002	Ireland	Laboratory observational study	ICUs isolates related to catheter infections	Microtiter plate assay and PCR detection of *ica* genes	*Staphylococcus* *epidermidis*
Paulitsch et al. [36]	2009	Austria	Observational microbiological study	Catheters from Hospitalized patients	Scanning electron microscopy	*Candida albicans*, *Candida glabrata*, *Candida parapsilosis*, *Candida tropicalis*
Perkins et al. [39]	2010	USA	Molecular observational study	Endotracheal tubes	qPCR and 16S rRNA sequencing	Oral microbiota including *Streptococcus*, *Prevotella*, *Neisseria*, and opportunistic pathogens
Djeribi et al. [26]	2012	Algeria France	Experimental microbiological study	Urinary catheters	SEM and crystal violet biofilm assay	*Pseudomonas* *aeruginosa*, *Acinetobacter* *baumannii*, *Klebsiella* spp.
Seddiki et al. [30]	2013	Algeria	Observational clinical study	Catheters in ICUs patients	Microscopy and SEM biofilm analysis	*Candida albicans*
Mirzaee et al. [28]	2014	Iran	Molecular observational study	ICUs isolates associated with devices	Microtiter plate assay and PCR detection of *ica*ABCD genes	*Staphylococcus aureus*
Kaur et al. [35]	2015	India	Prospective observational study	Central venous catheters	Tissue culture plate biofilm assay	*Staphylococcus aureus*, *Pseudomonas* *aeruginosa*, *Enterococcus faecalis*, *Acinetobacter* spp.
Abbes et al. [31]	2017	Tunisia	Experimental microbiological study	Catheter infection model	In vitro biofilm assays and in vivo catheter model	*Yarrowia lipolytica*, compared with *Candida* spp.
Patel et al. [25]	2021	India	Observational study	Urinary catheters (CAUTI)	Microtiter plate biofilm assay	*Escherichia coli*, *Klebsiella pneumoniae*, *Pseudomonas* *aeruginosa*, *Enterococcus faecium*, *Candida tropicalis*
Dewi et al. [33]	2021	Indonesia	Prospective observational study	Endotracheal tubes	Quantitative microtiter plate biofilm assay	Gram-negative pathogens associated with VAP
Medis et al. [29]	2023	Sri Lanka	Cross-sectional laboratory study	Central venous catheters	Phenotypic biofilm assays and antimicrobial susceptibility testing	Coagulase-negative staphylococci (*S. haemolyticus*, *S. epidermidis*)
Singh et al. [24]	2024	India	Observational study	Indwelling vascular catheters	Culture techniques and tube method biofilm assay	*Staphylococcus aureus*, CoNS, *E. coli*, *Klebsiella pneumoniae*, *Pseudomonas* *aeruginosa*
Mohamed et al. [32]	2024	Egypt	Prospective observational study	Urinary catheters	Microtiter plate assay and PCR detection of virulence genes	*Escherichia coli*

Abbreviations: CAUTI, catheter-associated urinary tract infection; CoNS, coagulase-negative staphylococci; ICUs, intensive care unit; PCR, polymerase chain reaction; qPCR, quantitative polymerase chain reaction; SEM, scanning electron microscopy; TEM, transmission electron microscopy; VAP, ventilator-associated pneumonia.

**Table 3:** Distribution of microorganisms by device type across included studies.

Device Type	Gram-Negative Bacteria	Gram-Positive Bacteria	Fungi	References
Endotracheal tubes	*Pseudomonas aeruginosa* *Acinetobacter baumannii* *Klebsiella* spp.	*Staphylococcus aureus*	*Candida* spp.	[33,34,37,39]
Vascular/central venous catheters	*Enterobacteriaceae* and other Gram-negative bacilli	*Staphylococcus aureus* *Staphylococcus epidermidis* *Coagulase-negative staphylococci* *Enterococcus* spp.	*Candida* spp.	[24,27,29,30,35]
Urinary catheters	*Escherichia coli* *Klebsiella* spp. *Proteus mirabilis* *Pseudomonas aeruginosa* *Acinetobacter baumannii*	*Enterococcus* spp.	*Candida tropicalis*	[25,26,32]
ICUs isolates or experimental models	Gram-negative bacteria	*Staphylococcus* spp.	*Yarrowia lipolytica* *Candida* spp.	[28,31,36,38]

**Table 4:** Biofilm detection methods used across included studies.

Method	Description	References
SEM/TEM (structural visualization)	Direct visualization of biofilm architecture on device surfaces	[26,27,30,34,36,37]
Microtiter plate assays (quantitative methods)	Quantitative or semi-quantitative assessment of biofilm formation using crystal violet or related assays	[25,28,29,31,33,35,38]
Molecular methods (PCR/gene detection)	Detection of biofilm-associated genes or virulence markers	[28,32,38]
Molecular profiling (16S rRNA sequencing)	Microbial community characterization	[39]
Culture-based and phenotypic methods	Microbial isolation, quantitative culture, or tube methods	[24,35]

## Data Availability

Data are contained within the article.

## Supplementary Materials

The following supporting information can be downloaded at: https://www.mdpi.com/article/10.3390/jcm15197388/s1, File S1: PRISMA-ScR Checklist.

## Abbreviations

CAUTI	Catheter-Associated Urinary Tract Infection
CDC	Centers for Disease Control and Prevention
CIED	Cardiac Implantable Electronic Device
CLABSI	Central Line-Associated Bloodstream Infection
CoNS	Coagulase-Negative Staphylococci
CRBSI	Catheter-Related Bloodstream Infection
CVC	Central Venous Catheter
EPS	Extracellular Polymeric Substances
HAIs	Healthcare-Associated Infections
ICUs	Intensive Care Units
PCC	Population–Concept–Context
PCR	Polymerase Chain Reaction
qPCR	Quantitative Polymerase Chain Reaction
rRNA	Ribosomal Ribonucleic Acid
SEM	Scanning Electron Microscopy
TEM	Transmission Electron Microscopy
VAP	Ventilator-Associated Pneumonia
